# A 20 gene model for predicting nodal involvement in bladder cancer patients with muscle invasive tumors

**DOI:** 10.1371/currents.RRN1248

**Published:** 2011-08-11

**Authors:** Garrett Dancik, Dara Aisner, Dan Theodorescu

**Affiliations:** ^*^Department of Surgery, University of Colorado, Aurora, Colorado, USA; ^†^Department of Pathology, University of Colorado, Aurora, Colorado, USA; ^‡^University of Colorado Cancer Center and ^§^The Public Library of Science (PLoS) is a non-profit open-access publisher, committed to making scientific and medical research literature a freely accessible public resource.

## Abstract

Bladder cancer is the fourth most common cancer in males worldwide and also the most expensive cancer to treat. Approximately 25% of patients with muscle invasive disease are found to harbor occult lymph node involvement at the time of cystectomy and this finding is associated with a 5-year survival rate of <30%. If these patients could be identified pre-operatively, use of neoadjuvant chemotherapy may be advantageous because this approach has been shown to confer a small survival advantage in patients with muscle invasive disease. However, because only a few patients benefit from this approach it has not been used extensively in the United States with fewer than 2% of patients undergoing this treatment. This is largely due to concerns that since neoadjuvant therapy is beneficial for only a few patients, it has the potential to delay surgery in the majority who do not benefit. However, since neoadjuvant therapy is most likely to benefit those patients at highest risk for progression of disease, it follows that patients with lymph node metastases would constitute an ideal group for such treatment. Hence, if patients with occult node involvement prior to cystectomy could be identified, they would constitute an ideal group for application of neoadjuvant therapy as they are most likely to benefit. In this summary, we describe the first multi-analyte gene expression model developed for predicting occult nodal involvement at cystectomy in bladder cancer patients, for the purpose of making better informed decisions regarding neoadjuvant therapy. The 20 gene model, which was developed on Affymetrix Human Genome U133A and U133 Plus 2.0 arrays, identified individuals with high relative risk (RR) of nodal involvement (RR = 1.74, 95% CI, 1.03 – 2.93) intermediate risk (RR = 1.05, 95% CI, .45 – 2.41), and low risk (RR = 0.74, 95% CI, 0.51 – 0.96), when evaluated in an independent test dataset. The 20 gene model can be applied to formalin-fixed paraffin embedded tissue with sufficient tumor content, making implementation in routine diagnostic tissue highly feasible. Although a clinical assay for the gene panel has not undergone analytic validation in a clinical laboratory setting, multiple platforms are available which could be utilized for routine testing, including real-time reverse transcriptase PCR directed against individual analytes as well as microarray approaches.

## **Background**

Approximately 20-30% of bladder cancer patients harbor muscle-invasive tumors at the time of diagnosis  [1] and the five year survival rate of these patients is approximately 50% [2]. Lymph node involvement is a strong indicator of poor prognosis, as evidenced in a long term study of over 1,000 patients treated by radical cystectomy and pelvic lymph node dissection, with or without adjuvant radiation or chemotherapy. Whereas the 5-year overall survival rate was 69% in patients with node negative tumors, the 5-year survival rate drops to 31% in patients with node positive tumors [3]. Several clinical trials [4] [5] and a meta-analysis [6] [7] analyzing outcomes in over 3,000 patients across 11 clinical trials have found that neoadjuvant chemotherapy confers a 5% absolute improvement in 5-year survival rates in patients with advanced bladder cancer. The clinical trial with the longest followup (8.7 years in the cystectomy only group and 8.4 years in the cystectomy + chemotherapy group) found that administration of neoadjuvant chemotherapy increased median survival (77 months vs. 46 months), according to an intention to treat analysis, conferred a 5% absolute improvement in 10-year survival rates, and decreased residual disease at the time of cystectomy (15% vs. 38% of patients) [5]. In addition, the benefit from chemotherapy is greater in patients with more invasive tumors (a 2.7 fold increase in median survival in patients with T3-T4a tumors vs. a 1.4 fold increase in median survival in patients with T2 tumors) [4], indicating that some patients are much more likely to benefit than others from neoadjuvant therapy. Because risk of nodal involvement increases with tumor stage [3], these patients are likely those with occult node positive disease. Additional advantages to neoadjuvant therapy include delivery during a time when micrometastatic disease is low, increased tolerance compared to adjuvant chemotherapy, and the potential to measure chemosensitivity [8]. However, a review of treatment patterns covering approximately 60% of all patients diagnosed with stage III bladder cancer between 1998-2003 found that only 1.2% of patients received neoadjuvant therapy [9], probably due to concerns of disease progression resulting from surgical delay, treatment side effects which may affect surgical outcomes, and overtreatment in patients without micrometastatic disease [10]. 

A test predicting nodal involvement prior to cystectomy would identify high risk patients likely to benefit from neoadjuvant therapy and low risk patients for which neoadjuvant therapy is unnecessarily aggressive and may negatively affect survival as cystectomy is delayed. There is currently one published gene expression model for quantifying the risk of nodal involvement in bladder cancer patients with muscle invasive tumors. This test is based on the expression profile of 20 genes and determines whether a patient has a “high” or “low” risk of nodal involvement. 

**Clinical Scenario**

The clinical situation in which this test is most likely to be beneficial is in the newly-diagnosed patient with muscle-invasive bladder carcinoma, with this test aiding in the decision to treat with neoadjuvant chemotherapy in those patients at high risk for nodal disease, versus the decision to proceed directly to surgical intervention in those with low risk of occult nodal metastasis.

**Test Description**

This gene expression test utilizes RNA extracted from either frozen or formalin-fixed paraffin-embedded (FFPE) tumor tissue obtained from transurethral resection (TUR), which is performed as a diagnostic procedure to determine depth of tumor invasion. The expression values for the twenty genes (21 microarray probes) in the signature is then obtained and a probability of lymph node involvement at cystectomy calculated. The test was developed and validated using gene expression data from Affymetrix Human Genome U133A and U133 Plus 2.0 arrays. A Bayesian weighted nearest neighbor prediction algorithm generates a posterior risk probability (*p*) of nodal involvement based on the Spearman’s rank correlation between a patient's 20-gene expression profile and the expression profiles from a training dataset containing patients with known nodal staging information. The risk probability is compared with a 23% baseline prevalence of nodal involvement in patients undergoing radical cystectomy [3] to classify a patient as high risk (*p* > .247), intermediate risk (.227 ≤ *p* ≤ .247) or low risk (*p* < .227). High, intermediate, and low risk groups have relative risks of 1.74, 1.05, and 0.74, respectively.

Utilization of this gene expression panel approach in a clinical setting requires extensive technical validation, and at least two approaches for clinical implementation can be envisioned.  The first is seen in several clinically available expression-based tests, including Oncotype DX^®^ (Genomic Health) [11], miRView™ mets and mets2 assays (Rosetta Genomics) [12], and CancerTYPE ID^® ^(bioTheranostics) [13] where each analyte is tested individually using a quantitative reverse-transcriptase qPCR (RT-PCR) approach.  This allows for more stringent control over the evaluation of each analyte, but is expensive and labor-intensive.  An alternative methodology is the clinical implementation of array-based technology, an approach which is increasingly embraced in clinical laboratories.  Although there is limited experience with issues related to technical validation, data interpretation and quality control of expression arrays in the clinical setting, two *in vitro* diagnostic multivariate index assays (IVD-MIA) utilizing microarray technology have been FDA approved for cancer diagnostics: the MammaPrint^TM^ microarray (Agendia) [14], which measures recurrence risk in breast cancer patients, and the Pathwork^®^ Tissue of Origin test (Pathwork Diagnostics) [15]. Regardless of the approach, technical validation requires determination of multiple test parameters, including accuracy, precision, analytic sensitivity, limit of quantification, and robustness as well as identification of standards for calibration and control of the test system.  

## **Public Health Importance**

Worldwide, bladder cancer is the fourth most common cancer and the ninth most common cause of cancer related deaths in males [2].  Lymph node involvement is a strong predictor of survival, however definitive evaluation of lymph node status can only be performed after the window for neoadjuvant therapy has elapsed.  A non-invasive modality by which patients most likely to harbor lymph node metastases can be identified has the potential to dramatically alter practice patterns with regard to implementation of neo-adjuvant therapy, and dramatically improve survival in a subset of patients.  As approximately 1/4 of patients with invasive bladder cancer have lymph node positive disease [3], the potential impact of such a 'personalized medicine' approach to treatment is substantial.

## **Published Reviews, Recommendations and Guidelines**

**Systematic evidence reviews** 

- The Cochrane Collaboration encourages the use of combination neoadjuvant therapy on the basis of a meta-analysis of over 3,000 invasive bladder cancer patients across 11 trials which found a statistically significant 5% absolute improvement in overall survival and 9% absolute improvement in disease free survival at 5 years associated with platinum-based combination chemotherapy [6] [7].  

**Recommendations by independent group** 

      The National Comprehensive Cancer Network (NCCN) [16] recommends considering neoadjuvant therapy for T3 bladder cancer patients, noting that a “modest survival benefit of neoadjuvant chemotherapy patients with muscle-invasive bladder cancer was noted in randomized trials and meta-analysis performed in patients receiving 3 cycles prior to cystectomy but not radiotherapy.”  

**Guidelines by professional groups**  

      The European Association of Urology (EAU) [8] notes that as “a result of a 5–8% overall survival (OS) advantage in recently published studies and meta-analyses, neoadjuvant cisplatin-containing combination chemotherapy should be considered and discussed with the patient in cases of muscle-invasive, clinically node-negative, and nonmetastatic (N0 M0) urinary bladder carcinoma, irrespective of definitive treatment.”

## **Evidence Overview**

***Analytic Validity*:** Test accuracy and reliability in measuring the gene expression profile for nodal involvement (analytic sensitivity and specificity).   

- The gene expression model was developed and validated using gene expression data profiled on Affymetrix GeneChip® Human Genome U133A and U133 Plus 2.0 arrays and processed by either Microarray Suite (MAS5) or Robust Multichip Average (RMA) algorithms [17].
- Affymetrix announced on May 9, 2011 that the U.S. Food and Drug Administration has cleared the addition of new gene expression reagents as accessories to its GeneChip® Microarray Instrument System for in vitro diagnostic (IVD) use (www.affymetrix.com). Affymetrix microarray platforms are already in use with two FDA-cleared tests, the Roche AmpliChip® CYP450 Test, which detects genetic variants for the metabolic genes CYP2D6 and CYP2C19, and Pathwork® Diagnostics’ Tissue of Origin Test which measures gene expression in > 2000 genes [15].
- One of the gene expression datasets used for model development was profiled from formalin-fixed, paraffin embedded (FFPE) tumor samples obtained from cystectomy. To ensure clinical applicability of the test to transurethral resection (TUR) specimens preserved by fresh freezing (FF), the 20 gene model utilizes 21 “high fidelity” probes whose expression values show strong correlation across FFPE and FF tissue preservation methods and whose expression values are similar between specimens obtained from cystectomy and TUR [17]. Specifically, a probe is considered "high fidelity" if the correlation in its expression values across 32 matched FFPE-FF samples was greater than 0 (p < 0.025, one-tailed test) and if its expression values were *not* significantly different (p< 0.01, two-tailed test) between 30 TUR specimens obtained from advanced Stage IV, node positive or metastatic patients and 25 cystectomies from node positive patients. The validation cohort was evaluated based on archival FFPE material.
- Although several studies have found that Affymetrix microarray platforms are reliable, on average, across all probes on the array [18], the reliability of the 21 probes in the gene model is currently not known. If implemented in a clinical laboratory setting, the analytic parameters of the test, regardless of platform, would be thoroughly evaluated.

***Clinical Validity*:** Test accuracy and reliability in identifying patients with nodal involvement (predictive value).

- The gene expression data sets used to develop and evaluate the model were obtained from patients profiled at the Memorial Sloan Kettering Cancer Center (MSKCC cohort, 66 patients) [19], patients profiled at l'Hôpital de l'Hôtel-Dieu at Laval University, Québec, Canada (Laval Cohort, 188 patients), and patients from a phase III, multicenter randomized controlled trial comparing two adjuvant chemotherapy regimens [20] (AUO-AB 05/95, AUO cohort, 185 patients). The MSKCC and Laval cohorts were used for training while the AUO cohort was used for independent validation. Given the potential bias of gene profiling studies performed on small specimen sets, the large training and validation sets utilized in this study confer additional substantiation of the clinical utility of this assay.
- Model evaluation was carried out using Receiver Operating Characteristic (ROC) curve analysis.  The area under the curve (AUC) is 0.72 and 0.67 in the training and validation datasets, respectively [17].
- The thresholds were established based on the training set for distinguishing high, intermediate, and low risk groups, with relative risks of 2.25 (95% CI, 1.18-4.28), 1.02 (95% CI, 0.46 – 2.26), and 0.47 (95% CI, 0.31, 0.70), respectively. When applied to the independent validation set, these thresholds demonstrated relative risks of 1.74, (95% CI, 1.03 – 2.93) 1.05 (95% CI, .45 – 2.41), and 0.74 (95% CI, 0.51 – 0.96), for high, intermediate and low risk groups respectively, demonstrating a high degree of concordance between the training and validation sets [17].
- The authors have provided us with the predicted risk scores of the patients in their validation dataset, allowing us to calculate additional performance measures. Using the recommended high and low risk cutoffs [17], in the validation dataset the model has a sensitivity for nodal involvement of 0.44, a specificity of 0.70, a positive predictive value (PPV) of  0.30, and a negative predictive value (NPV) of 0.81. PPV and NPV calculations assume a 23% prevalence of nodal involvement at time of cystectomy [3]. Importantly, if patients with higher stage disease were to be selected for testing, these characteristics would change. For example, if only T3 patients were selected, the prevalence of occult nodal disease in these patients would be 39% [3]. Hence the PPV and NPV in this scenario would be 0.48 and 0.66, respectively.

***Clinical Utility*: **Net benefit of test in improving health outcomes.

      The gene expression model was validated in a prospectively collected cohort of patients enrolled in a phase III clinical trial [20], indicating that the gene model can accurately identify high and low risk patients in clinically relevant patient populations [17]. However, prospective studies are needed to fully define the clinical utility of the 20 gene node signature and to assess whether neoadjuvant chemotherapy confers a larger survival benefit in the high risk patients identified by this test. 

**Limitations**

There is currently only a single peer-reviewed publication describing the development and validation of the genomic test.  The testing is not currently available in a clinical laboratory setting. 

**Conclusions**

The 20 gene model described in this summary is the first published model for predicting nodal involvement in bladder cancer patients and by providing this molecular intelligence, has the potential to strategically increase the use of neoadjuvant therapy and increase bladder cancer survival rates while limiting unnecessary aggressive treatment. Importantly, the model was developed with clinical applicability in mind. Specifically, the 20 genes (21 probes) used in the model are reliably expressed in TUR specimens and can be reliably detected with both FF and FFPE preservation methods, the clinical conditions under which the model will be employed and allowing for retrospective analysis of archival tissue after the patients has undergone TUR in the course of routine clinical care and muscle invasive disease discovered. Although the model has been validated in prospectively collected samples from an independent clinical trial, further refinement is needed in the clinical laboratory setting to bring this test to routine clinical use. Nevertheless, the development of the model is an important milestone on the path to the personalized treatment of bladder cancer.

**Links**

None identified

Last updated: August 4, 2011

## **Funding information**

This work was supported by National Institutes of Health grant CA104106 to D.T. 

## **Competing interests**

GD and DT participated in the development and validation of the gene model and are coauthors on the publication describing its development and validation [17]. IP rights on this test have been filed at the University of Colorado.
